# By Their Fruits Ye Shall Know Them

**Published:** 1882-01

**Authors:** 


### Editorial.

---

### "By Their Fruits ye Shall Know Them."

The necessity for a higher standard of medical education has long been recognized by the enlightened and progressive members of the profession, and a steady advance in this direction is the outgrowth of the discussions, which, during the past decade, have occupied so much of the time and attention of medical societies. This movement for a more thorough preparation of the men who are sent forth from our medical colleges, duly invested with the legal authority to practice medicine and surgery, has arisen from the necessity that with the growth of the country in wealth and population, the standard of the learned professions should keep apace with the progressive ideas, prevailing in social as well as in scientific circles. The public also have recognized, in common with the profession, the dangerous consequences of the laxity, heretofore prevailing in the education of those to whom their sanitary interests are intrusted. The pressure, therefore, from without, and the plain defects acknowledged to exist within the profession, have begun to yield fruits through the advanced curriculum, adopted at Harvard and several of our leading medical schools.

We refer to this subject at this time, because circumstances have of late arisen in this city, which demonstrate forcibly the disastrous effects of a disregard of the interests of the community in the character and the professional attainments of those sent forth to guard and protect its sanitary interests. A better illustration of the danger to communities of empowering irresponsible parties with authority to determine the qualifications of candidates for the degree of Doctor of Medicine, has rarely been afforded than is at present witnessed in the northern portion of this city, in which prevails an endemic of Variola, arising from one or more cases of this loathsome disease, which had passed through the successive stages until convalescence was well assured, without being diagnosed by the practitioner, to whom the medical care of the patients was committed. One of these cases had been sick nearly three weeks, and during this period had exposed the entire neighborhood which was in ignorance of the true character of the disease with which they were surrounded. These patients were under the care of one Von Schulenberg, who holds a degree from the defunct College of Physicians and Surgeons in this city, the charter of which, by the late decision of the Supreme Court, was declared null and void.

We have heretofore refrained from any criticism of this institution, or reference to the real dangers to the public, to which its establishment and continuance have given rise. This illustration of the ignorance of one of its graduates more forcibly demonstrates its true character than any argument we could present. It justifies the action of the county society in taking steps to test the validity of its charter, which happily for the public resulted in closing its doors. It exposes the true character and motives of its faculty in turning loose upon the community, under the assumed authority of its illegal diplomas, ignorant men, to swell the army of imposters and quacks who play upon the credulity of the public, often with more fatal consequences than is experienced in the present endemic. In the minds of the community at large, it depreciates the high and noble purposes of the science of medicine, through the lamentable deficiencies of those who are mere pretenders, without either legal, professional or intellectual qualifications for its duties.

While the profession have been alive to the dangers which were surely to flow from such a source, the law in its operations has been slow to interpose its authority, and serious mischief has been perpetrated through which human life is sacrificed, and the legal safeguards upon which the public depend for its protection against infectious diseases, made of no effect. It is only a pestilence which devastates communities and scatters death upon every side, that awakens a realizing sense of prevailing dangers. This instance shows that there are agencies requiring the strong arm of the law, and the force of an enlightened public sentiment for their control. We hope this, among many others which we could mention, may serve to elucidate the need of higher medical education, and the abolition of medical schools which, with sinister motives, foster professional ambition at the expense of human life.

It is only necessary to direct attention to the wanton ignorance of the pretender through whom an entire community has been needlessly exposed to one of the most loathsome of the infectious diseases, to enable the profession and the public to trace the responsibility to its rightful source; while we may entertain the hope, that, through such experiences a higher appreciation of legitimate medicine may result, and the dependence of the public upon its skill and knowledge in the guardianship of the public health more fully demonstrated and acknowledged.

